# Metallothionein-II Inhibits Lipid Peroxidation and Improves Functional Recovery after Transient Brain Ischemia and Reperfusion in Rats

**DOI:** 10.1155/2014/436429

**Published:** 2014-02-25

**Authors:** Araceli Diaz-Ruiz, Patricia Vacio-Adame, Antonio Monroy-Noyola, Marisela Méndez-Armenta, Alma Ortiz-Plata, Sergio Montes, Camilo Rios

**Affiliations:** ^1^Departamento de Neuroquímica, Instituto Nacional de Neurología y Neurocirugía Manuel Velasco Suarez, Avenida Insurgentes Sur No. 3877, 14269 México City, DF, Mexico; ^2^Laboratorio de Neuroprotección, Facultad de Farmacia, Universidad Autónoma del Estado de Morelos, Mexico; ^3^Laboratorio de Neuropatología, Instituto Nacional de Neurología y Neurocirugía Manuel Velasco Suarez, Mexico; ^4^Departamento de Sistemas Biológicos de la Universidad Autónoma Metropolitana, Unidad Xochimilco México, Mexico

## Abstract

After transient cerebral ischemia and reperfusion (I/R), damaging mechanisms, such as excitotoxicity and oxidative stress, lead to irreversible neurological deficits. The induction of metallothionein-II (MT-II) protein is an endogenous mechanism after I/R. Our aim was to evaluate the neuroprotective effect of MT-II after I/R in rats. Male Wistar rats were transiently occluded at the middle cerebral artery for 2 h, followed by reperfusion. Rats received either MT (10 **μ**g per rat i.p.) or vehicle after ischemia. Lipid peroxidation (LP) was measured 22 h after reperfusion in frontal cortex and hippocampus; also, neurological deficit was evaluated after ischemia, using the Longa scoring scale. Infarction area was analyzed 72 hours after ischemia. Results showed increased LP in frontal cortex (30.7%) and hippocampus (26.4%), as compared to control group; this effect was fully reversed by MT treatment. Likewise, we also observed a diminished neurological deficit assessed by the Longa scale in those animals treated with MT compared to control group values. The MT-treated group showed a significant (*P* < 0.05) reduction of 39.9% in the infarction area, only at the level of hippocampus, as compared to control group. Results suggest that MT-II may be a novel neuroprotective treatment to prevent ischemia injury.

## 1. Introduction

Stroke is a disabling condition with devastating consequences for patients. Worldwide, it accounts for approximately 5.5 million deaths annually, with 44 million disability-adjusted life-years lost. As associated to aging, the prevalence of stroke is expected to increase significantly around the world [1]. This condition, in addition to the serious health complications, generates high costs; thus, health care for stroke survivors has been estimated to be $18.8 billion dollars in 2008. The loss of productivity and premature mortality are estimated at an additional cost of $15.5 billion [2].

After transient cerebral ischemia and reperfusion (I/R), damaging events initiate as result of the suppression of energy production caused by the interruption of oxygen and glucose supply to brain. Excitotoxicity is the leading mechanism of damage as a consequence of an increased release of excitatory neurotransmitters, such as glutamate, into the extracellular space [3]. Oxidative damage also participates in the acute phase after I/R and is caused by excessive amounts of reactive oxygen and nitrogen present in nervous tissue [4]. Inflammatory response is initiated as a result of the blood-brain barrier breakdown [5]; those events trigger apoptosis [6]. Oxidative stress is an important mechanism involved in this process, as the antioxidant defenses are upregulated in order to cope with the reactive oxygen (ROS) and nitrogen species. All these free radicals damage the membrane's fatty acids through a deleterious process known as lipid peroxidation (LP) [7].

Metallothionein (MT), on the other hand, is an antioxidant thiol defense after IR. Metallothioneins are a family of low-molecular-weight (6–7 kDa) proteins with high content of cysteine residues and bound metal ions. The metal thiolate clusters (Scys-M-Scys) exist in two separate globular domains, which are linked by a small lysine-rich region; the domain in the C-terminus contains 11 cysteine residues and is able to bind four divalent or six monovalent metals, while the *N*-terminal domain contains 9 cysteine residues capable of binding three divalent or six monovalent metals [8]. MT functions include transport and storage of essential transition metals, detoxification and protection against ROS, which are important mechanisms for host defense response, immunoregulation, cell survival, and brain repair [9]. MT-III has been located abundantly in neuronal cell bodies in CA1-3 regions of hippocampus, dentate gyrus, cerebral cortex, olfactory bulb, and Purkinje cells in cerebellum [10]. However, the role of MT-III in neuroprotection is controversial, since there are reports showing its neuroprotective effect by preventing reactive oxygen species (ROS) formation, increasing the expression of heme oxygenase-1 [11]. Also, MT-III has been described to inhibit neurite outgrowth and to promote neuronal death [12]. On the other hand, MT-I and MT-II are expressed in astrocytes and microglia as well as in monocytes/macrophages [13]. The participation of MT as a neuroprotective mechanism in cerebral ischemia has been shown in MT-I and MT-II knockout (KO) mice submitted to permanent middle cerebral artery occlusion (MCAO) [14]. MT-I and MT-II KO mice showed greater neuronal damage, as compared to wild-type mice. The exogenous administration of MT-II has shown to inhibit neuronal damage in a model of autoimmune encephalomyelitis in rats [15] and in a spinal cord injury model [16]. Based on this information, we tested the ability of exogenously administered MT-II to prevent I/R induced brain damage in rats.

## 2. Materials and Methods

### 2.1. Animals

Male Wistar rats weighing 250–300 g were maintained under standard laboratory conditions and had free access to food and water. The protocols for animal use were approved by the Animals Ethics Committee of the National Institute of Neurology and Neurosurgery of Mexico.

### 2.2. Surgery

We used the MCA occlusion experimental model of focal cerebral ischemia reported by Longa et al. [17] modified to achieve reperfusion 2 h after ischemia. Animals were anesthetized with 3% halothane using a facemask. Body temperature was maintained at 37°C with a warm pad during the surgical procedure and afterward until the recovery of rats from anesthesia. A longitudinal incision was made in the middle of the ventral cervical skin. The right common carotid artery, right internal carotid artery, and right external carotid artery were exposed. The distal portion of the right external carotid artery was then ligated and cut. A nylon suture (3–0) was introduced into the lumen of the right external carotid toward the internal carotid. The suture was advanced 17 mm into the right internal carotid and left there. Finally, the incision was closed and left under controlled conditions for 2 h. After this time, the reperfusion started by opening the wound to retract the filament, which was pulled out completely. Immediately after reperfusion, rats were evaluated for neurological deficits using the scale described by Longa et al. [17].

### 2.3. Pharmacological Treatments

Rats were randomly allocated into four groups as follows: group 1: sham operation plus vehicle, group 2: two hours of ischemia and reperfusion plus vehicle (saline solution, 0.9% NaCl), and group 3: two hours of ischemia and reperfusion exogenously administered with 2 i.p. doses of 10 *μ*g per rat MT-II dissolved in saline solution (metallothionein-II from rabbit liver, Sigma M9542), according to Arellano-Ruiz et al. [16]. MT-II was selected on the basis of the stability of the protein. MT-II half-life in adult animals is 21–33 hours, while the half-life of MT-I is shorter. This may indicate that MT-I is more susceptible to degradation than MT-II [18]. All animals received two doses of vehicle or MT at 30 min and 8 hr after ischemia. These times were chosen on the basis of the therapeutic window for Stroke.

### 2.4. Reagents Lipid Peroxidation Assay

Quinine 90% was purchased from Sigma Aldrich; chloroform and methanol of HPLC grade were purchased from Merck Chemicals.

### 2.5. Lipid Peroxidation Assay

Lipid fluorescence products' formation was measured after ischemia and reperfusion by using the technique described by Triggs and Willmore [19], modified by Santamaria and Rios [20]. All animals were sacrificed 24 hr after ischemia, the time of peak levels of lipid peroxidation (LP) reported by Thiyagarajan and Shrama [21]. Rats were killed by decapitation, and their frontal cortex and hippocampus (both ipsilateral and contralateral to the injury) were dissected out, according to Iversen and Glowinski [22]. Tissues were weighed and homogenized in 3 mL of cold 0.9% NaCl solution. One-milliliter aliquots from the homogenate were added to 4 mL of a chloroform-methanol mixture (2 : 1 *v/v*). After stirring, the mixture was ice-cooled for 30 min to allow phase separation and the fluorescence of the chloroform layer was measured in a Perkin-Elmer LS50B Luminescence spectrophotometer at 370 nm of excitation and 430 nm emission wavelengths. The sensitivity of the spectrophotometer was adjusted to 150 units of fluorescence with a quinine standard solution (0.1 g/mL). Results were expressed as fluorescence units/g of wet tissue.

### 2.6. Behavioral Assessment

In this study, we determine neurological deficit using the functional scale described by Longa et al. [17]. This scale was standardized specifically for the model of ischemia/reperfusion used, and injury is functionally evaluated mainly as motor deficits present after damage. Brain regional-specific functional alterations were not evaluated here, as we expected a more general protection with the treatments. All animals were evaluated 2, 4, 24, 48, and 72 hours after reperfusion to record the presence or absence of neurological signs in rats as follows: 0: no observable deficit, 1: forelimb flexion, 2: unidirectional circling, 3: falling to the contralateral injury side, 4: decreased level or lack of consciousness, and 5: death after surgery. All animals were allocated in individual acrylic cages with sterile sawdust and received food and water ad libitum.

### 2.7. Morphometric Analysis at the Level of Hippocampus

Seventy-two hours after ischemia, all animals were anesthetized by i.p. injection of pentobarbital. Then, 1 mL of heparin was administered, and rats were perfused transcardially with 10% buffered formalin. The brains were removed from the skull cut with a matrix (coronal rodent brain matrix, EMS) at 2 mm thick and embedded in paraffin. Coronary sections 10 *μ*m thick were obtained containing cortex and hippocampus. Those regions were selected using the Paxinos and Watson stereotaxic atlas (Bregma −2.40 to Bregma −2.64) [23]. After staining with hematoxylin-eosin, the area of ischemia was determined, according to the method described by Niyaz et al. [24]. Tissue slides (one per rat) were digitized using a computerized system equipped with IM500 software and a 300 FX digital camera. Area analysis was performed with an Image Database V.4.01 (Leica) and a CCD-IRIS Sony camera, using morphometric assessment. All histological preparations were assessed by a pathologist blind to the treatments. Results were expressed as percentage of tissue damage of cortical tissue.

### 2.8. Statistical Analysis

Results of LP are expressed as mean ± S.E.M. Statistical significance between groups was determined by analysis of variance, followed by Tukey's test, after testing for homogeneity of variances. Statistical significance between contralateral and ipsilateral cortex and hippocampus values was determined using paired *t*-test.

Significant differences in Longa scores were determined using the repeated-measures ANOVA, followed by Dunnett's test. Finally, the results of morphometric data were analyzed using Student's *t*-test for independent samples. All statistical analyses were performed using the SPSS 19.0 software.

## 3. Results

### 3.1. Metallothionein II Inhibits Lipid Peroxidation in Frontal Cortex and Hippocampus after Transient Cerebral I/R in Rats

**Figure 1:**
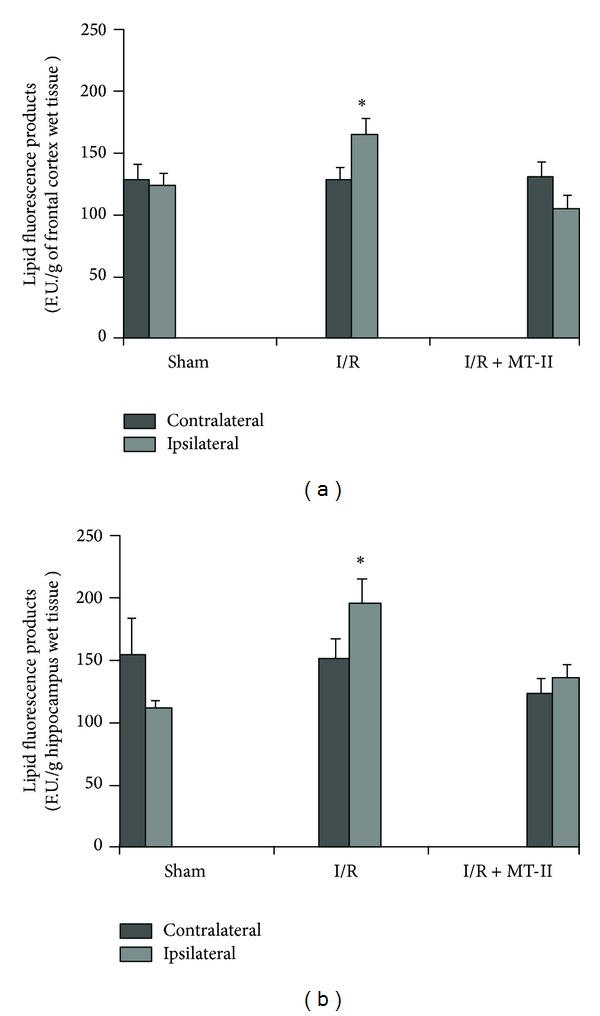
Lipid peroxidation 24 h after transient cerebral ischemia and reperfusion (I/R). Sham: rats without I/R; I/R: rats with 2 h of ischemia and 22 of reperfusion plus vehicle; I/R + MT-II: animals with 2 h of ischemia and 22 of reperfusion treated with MT-II (10 *μ*g/per rat) 30 min and 8 h after ischemia. (a) Lipid peroxidation in frontal cortex and (b) Lipid peroxidation in hippocampus. The results are expressed as means ± S.E.M. of 8–10 animals per group. *Different from both sham group and contralateral striatum (*P* < 0.05). One-way ANOVA followed by Dunnett's test for between-groups comparison and paired-samples *t*-test for contralateral versus ipsilateral comparisons.

Figure 1 shows the effect of exogenously administered metallothionein-II on LP assessed 24 h after ischemia, which was evaluated both on the contralateral side as in the ipsilateral side of I/R. Figure 1(a) shows the mean ± one S.E.M. of 7 to 10 animals per group tested, measured in the frontal cortex, and Figure 1(b) shows the results obtained in the hippocampus. The results are expressed in fluorescence units per gram of wet tissue (UF/g wet tissue).

LP values at frontal cortex level in the contralateral side were 140.81 ± 13.71, 136.83 ± 8.76, and 131.56 ± 14.46 for sham group, I/R group, and I/R+MT-II group, respectively. As observed, the average values are similar in all groups without significant differences. While the ipsilateral side is damaged, data observed were 136.41 ± 15.38, 178.27 ± 17.35, and 112.14 ± 13.35 for the sham group, I/R group, and I/RM-II group, respectively, showing a significant increase in the LP by I/R of 30.7%, when compared to the sham group. Treatment with MT-II decreased those values to the levels of the sham group. This reduction was statistically significant (*P < 0.05).

Likewise, the LP data in the contralateral side to I/R hippocampus showed similar results in all groups (162.29 ± 22.55, 170.02 ± 18.44, and 143.14 ± 26.33 for the sham group, I/R group, and I/RMT-II group, resp.), while the values on the ipsilateral side, LP indicates baseline levels in the sham group of 165.8 ± 16.26 which are lower than those observed in the tissue by the effect of I/R (209.58 ± 17.95) that were statistically different (*P* < 0.05). Again, MT-II treated rats showed diminished values (I/RMT-II group) of 136.44 ± 11.61 when compared to I/R group (*P* < 0.05).

### 3.2. Metallothionein-II Treatment Reduces Neurological Deficits after Transient Cerebral I/R in Rats

**Figure 2:**
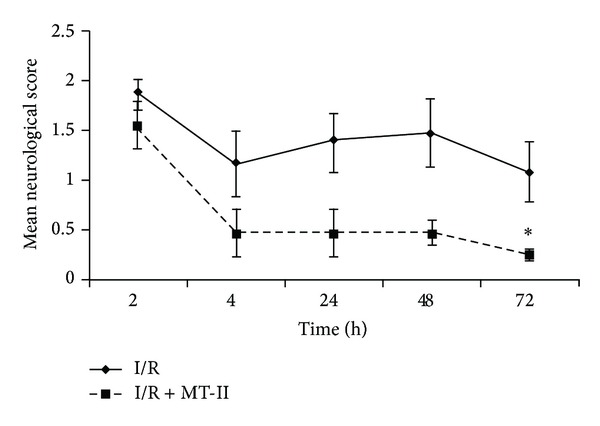
Neurological deficits evaluated after transient cerebral ischemia and reperfusion (I/R) at several times after administration of MT-II. Rats receiving 250 *μ*L of saline solution as vehicle (I/R) or MT-II(I/R + MT-II) at a dose of 10 *μ*g per rat via i.p. dissolved in 250 *μ*L of saline solution. Both treatments were at 30 min and 8 hours after ischemia and neurological deficits were evaluated with Longa scale at 2, 24, 48, and 72 h. Results are expressed as the mean ± S.E.M. of scores of *n* = 8–10 animals per group. * Different from control group, *P* < 0.05, repeated-measures ANOVA, followed by Dunnett's test.

Figure 2 shows the effect of MT-II treatment on functional recovery after cerebral I/R assessed by the Longa scale. High scores in these neurological scales correspond to a greater neurological deficit. The global neurological test scale provides a more general indication of neurological differences between control (I/R) and I/R MT-II groups. The mean values of I/R group animals were 1.85 ± 0.15 at 2 h and 1.08 ± 0.3 at 72 hours after ischemia. These results indicate little recovery of the animals. Meanwhile, animals in the group I/R plus MT-II showed mean values of 1.54 ± 0.24 at 2 h and of 0.23 ± 0.06 at 72 h after ischemia (*P* < 0.05). When comparing functional recovery of animals at the end of the study (72 hours), we observed a greater functional recovery of 78.7% in the group of animals receiving I/R plus MT-II, as compared to animals receiving I/R only; this difference was statistically significant (*P* < 0.05).

### 3.3. Metallothionein-II Diminished the Damaged Area after Transient Cerebral Ischemia and Reperfusion

**Figure 3:**
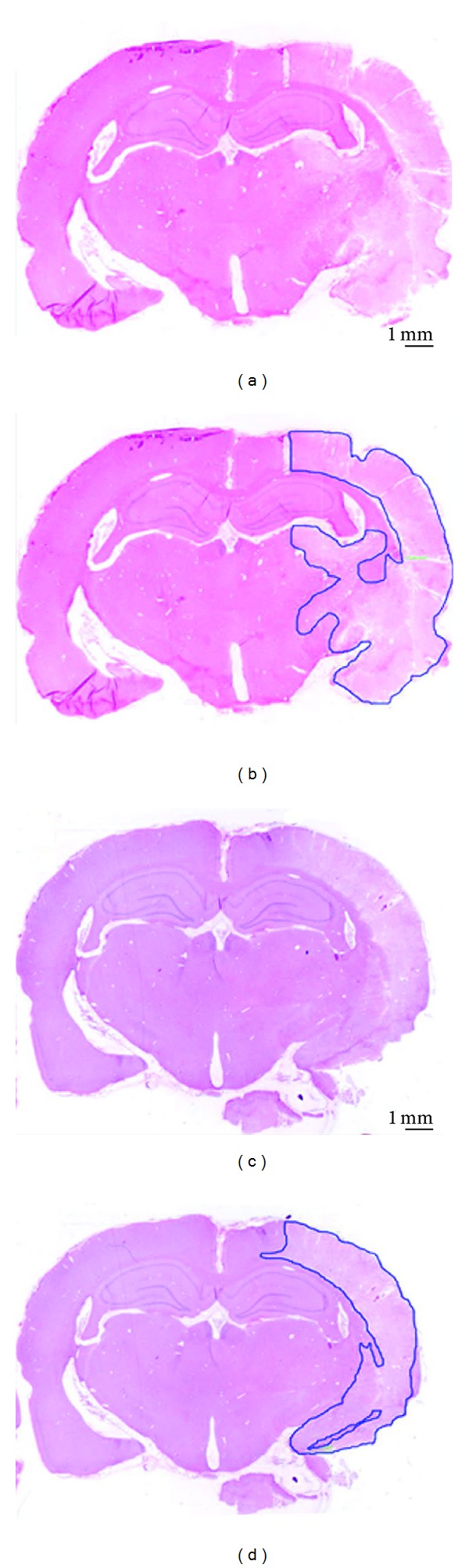
Representative photomicrographs at hippocampus level of transient cerebral ischemia and reperfusion (I/R) evaluated 72 h after damage. (a) and (b) are from animals with I/R treated with 250 *μ*L of saline solution (I/R). (c) and (d) are from rats submitted to I/R plus MT-II. Both groups were administered at 30 min and 8 h after cerebral ischemia. (a) and (c) brain sections without specifying the area. (b) and (d) infarct area in mm^2^. Hematoxylin-eosin staining was applied. Scale bars = 1 mm.

**Figure 4:**
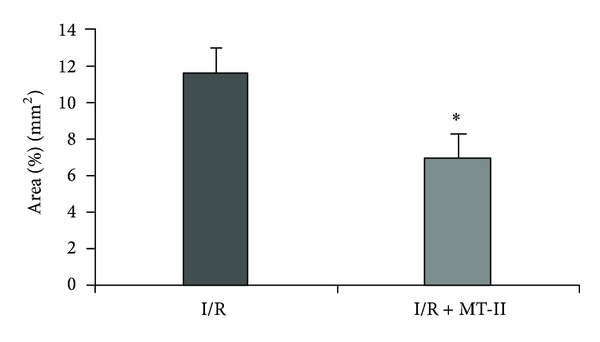
Percentage of tissue area damaged at the level of hippocampus evaluated 72 hours after transient cerebral ischemia and reperfusion. I/R: animals with ischemia and treated with a vehicle; I/R + MT animals with ischemia and treatment with MT-II at a dose of 10 *μ*g per rat dissolved in 250 *μ*L of saline solution i.p. The results are given in percentage of tissue damage with respect to 100% of brain tissue assessed ± S.E.M. of 8–10 animals per group. *P < 0.05, Student's *t*-test for independent samples.

Figure 3 shows representative photomicrographs of transient cerebral ischemia and reperfusion evaluated at the level of hippocampus. As clearly seen, there is a greater amount of damaged tissue in animals receiving I/R plus vehicle, when compared with the group of animals receiving I/R plus MT-II treatment. Likewise, Figure 4 shows the quantitation of the tissue damage performed 72 hours after I/R. Values are given in percentage of tissue damage with respect to 100% of brain tissue in the slide. The amount of tissue damage in animals receiving I/R and treated with vehicle was 11.58 ± 1.39, as compared to values from the group receiving I/R plus MT-II treatment (6.96 ± 1.23); this difference was statistically significant (*P* < 0.05).

## 4. Discussion 

In the present work, we demonstrated that the administration of exogenous MT-II decreases oxidative damage, evaluated as the production of lipid fluorescent products (LP), both in hippocampus and frontal cortex. In addition, this treatment promoted functional recovery and reduced the amount of damaged tissue after transient cerebral I/R in rats, but only at the level of hippocampus. The antioxidant effect of MTI/II also has been tested in primary cortical neuron/astrocyte cultures from neonatal MT-I/II deficient (MT^−/−^) and wild-type (MT^+/+^) mice against NMDA-mediated injury. The findings showed that MT-I/II expression was increased by NMDA in MT^+/+^ cultures but was not detectable in MT^−/−^ cultures. NMDA concentration dependently induced oxidative injury in both MT^+/+^ and MT^−/−^ cultures as evidenced by decreased cell viability, increased lipid peroxidation, and DNA damage. However, these toxic effects were greater in MT^−/−^ than values of MT^+/+^ cultures. NMDA significantly increased reactive oxygen species (ROS) generation and disrupted mitochondrial membrane potential in neurons in MT^+/+^ cultures, and these effects were exacerbated in MT^−/−^ cultures; these results showed that basal MT-I/II provides protection against NMDA-mediated oxidative injury [25]. It is important to note the relationship between oxidative damage mediated by the activation of NMDA-R and neuroprotection exerted by MT-I/II, as both mechanisms (excitotoxicity and oxidative stress) are remarkably important in the acute phase after I/R.

The neuroprotective effect of MT-I/II has been observed in several models of brain damage. Trendelenburg et al. [13] applied serial analysis of gene expression to study differentially expressed genes in mice brains 14 hours after the induction of focal cerebral ischemia and demonstrated that metallothionein-II (MT-II) was the most significantly upregulated transcript in the ischemic hemisphere. That upregulation of both MT-I and MT-II was confirmed by Northern blotting. MT-I and MT-II mRNA expression increased as early as 2 hours after transient ischemia, with a maximum after 16 hours. Likewise, an immunohistochemistry study revealed that MT-I/-II is localized in astrocytes as well as in monocytes/macrophages [13]. Taken together, all those results pointed out the neuroprotective role of metallothioneins in ischemic damage of the brain. Finally, Prado et al. [26] demonstrated that presence of MT-I/II after induction of cortical cryolesion in wild-type and MT-I/II deficient mice is responsible for neuroprotection; the authors examined the effect of administration of the selective phosphodiesterase-5 inhibitor sildenafil (10 mg/kg) 2 h before and 24 and 48 h after damage. The results showed that, in wild-type animals, sildenafil induces similar changes in glial reactivity, angiogenesis, and antioxidant and antiapoptotic effects in the cryolesioned cortex as those observed in rats with Zaprinast (nonselective cGMP-cyclic nucleotide phosphodiesterase inhibitor), indicating that inhibition of PDE5 is responsible for the neuroprotective actions. However, these effects were not observed in MT-I/II deficient mice. Likewise, they showed that sildenafil significantly increases MT-I/II protein levels in the lesioned cortex and MT-I/II immunostaining in glial cells around the lesion. Taken together, these results indicate that cGMP-mediated pathways regulate expression of MT-I/II and support the involvement of these proteins in the neuroprotective effects of sildenafil in focal brain lesions. Some other authors have found a lack of neuroprotection against I/R-induced damage of MT-I and MT-II null mice; however, they used a neonatal model of damage, and, thus, the inherent differences between developing and mature brains may account for the lack of protection observed by authors [27]. There is strong evidence of the neuroprotective effect of MT-II in various models of nervous system damage when its expression is induced; however, there is scarce information regarding the pharmacological use of exogenously administered MT. An interesting finding of the present study is that, only at the level of hippocampus, among all other brain regions examined, showed a significant protection by MT-II, as observed by histological markers (Figure 3). This is probably due to the abundance of zinc and MT-II basal expression in that brain region [28]. The mechanism through which MT-II exerts its protective effect when exogenously administered has not been elucidated; however, there is evidence that this protein can be carried by the megalin receptor, a known multiligand, endocytic receptor with significant physiological function. It is expressed primarily in polarized epithelial cells and, with a few exceptions, it is located in the apical membranes, with a molecular weight 6kDa; it is expressed in the choroid plexus ependymal cells that line the cerebral ventricles in central nervous system [29]. Recently, Lewis et al. [30] analyzed the distribution of MT-IIA when administered exogenously by intraperitoneal or intramuscular injections in metallothionein deficient mice. The results showed that MT-IIA was detected within epithelial cells of the kidney cortical and medullary tubules within 1 h of either intramuscular or intraperitoneal injection. Additionally, MT-IIA was detected in the urine 1 h after injection, indicating a rapid absorption into the circulation and filtration through the kidney glomerulus. A portion of the intramuscularly injected MT-IIA remained within the muscle for at least 24 hours after injection. No MT-IIA was observed in the liver nor in the brain after either a single injection or a series of MT-IIA injections. This is probably due to the exclusion of MT-IIA through the intact blood-brain barrier (BBB), although a receptor for MT-I/II (megalin) is present in the choroid plexus. In our model of I/R, BBB is broken and remains permeable to blood products for several hours [31]. The neuroprotective effect of exogenously MT-I/II given intraperitoneally to rats in experimental models where BBB is decreased [9, 15], like I/R, offers a pharmacological opportunity to test the neuroprotective abilities of the protein.
